# Fruquintinib Enhances the Antitumor Immune Responses of Anti-Programmed Death Receptor-1 in Colorectal Cancer

**DOI:** 10.3389/fonc.2022.841977

**Published:** 2022-03-17

**Authors:** Qingli Li, Xiaojiao Cheng, Cong Zhou, Yao Tang, Fuli Li, Baiwen Zhang, Tinglei Huang, Jianzheng Wang, Shuiping Tu

**Affiliations:** ^1^State Key Laboratory of Oncogenesis and Related Genes, Department of Oncology, Renji Hospital, School of Medicine, Shanghai Jiao Tong University, Shanghai, China; ^2^Department of Oncology, Henan Cancer Hospital, The Affiliated Cancer Hospital of Zhengzhou University, Zhengzhou, China

**Keywords:** fruquintinib, sintilimab, immunotherapy, microsatellite stable colorectal cancer, anti-angiogenesis

## Abstract

**Background:**

Programmed death receptor-1 (PD-1) blockade shows little benefit in patients with microsatellite-stable colorectal cancer (MSS-CRC). Fruquintinib is a China-made anti-angiogenic drug which is approved for the third line therapy in mCRC. This study investigates the effect of the combination of fruquintinib and PD-1 blockade on MSS-CRC and its relative mechanisms.

**Methods:**

The mouse allograft tumor models that represent MSS and microsatellite instability (MSI) CRC were established using murine CT26 and MC38 colon cancer cells, respectively, to assess the treatment efficacy. The percentages of immune cells were detected in the peripheral blood, spleen and tumor tissues in the tumor-bearing mice by flow cytometry analysis. Angiogenesis in tumor tissues was detected by immunofluorescence. The safety of drug treatment was evaluated by histopathology analysis in murine main organs. The efficacy of the combination of fruquintinib and sintilimab were verified in the treatment of MSS-CRC patients.

**Results:**

Our results showed that the combination of fruquintinib and sintilimab exhibited the strongest inhibition of tumor growth and achieved the longest survival time in mice bearing MC38 or CT26 xenograft tumors, compared to fruquintinib and sintilimab alone. Mechanistically, the combination of fruquintinib and sintilimab reduced angiogenesis, reprogramed the vascular structure, enhanced the infiltration of CD8^+^T cells (p<0.05), CD8^+^TNFα^+^ (p<0.05) T cells and CD8^+^IFNγ^+^ (p<0.05) T cells and reduced the ratios of MDSCs and macrophages in mice. There was no obvious toxicity observed in the main organs of the tumor-bearing mice with the combined treatment. Moreover, the treatment using the combination of fruquintinib and sintilimab achieved effective response in five patients with refractory advanced MSS CRC.

**Conclusion:**

Our results show that the combination of fruquintinib and sintilimab greatly inhibits CRC growth by altering tumor immune microenvironment. This study provides the rational for using the combination of fruquintinib and anti-PD-1 antibody for the treatment of advanced CRC.

## Introduction

Colorectal cancer is the second most common cause of cancer-related death worldwide (1).The latest research results have showed that the use of pembrolizumab as the first-line therapy for metastatic colorectal cancer (mCRC) with high microsatellite instability (MSI-H) or deficiency mismatch repair (dMMR) gene few treatment-related adverse events (2). Nivolumab plus ipilimumab demonstrated high response rates, promoting progression-free survival, manageable safety, and meaningful improvements in patients with dMMR/MSI-H mCRC (3). Although immunotherapy has achieved significant effects on advanced colon cancer- MSI-H or dMMR (4–6), the therapy is limited in 80%-90% advanced colon cancer patients-with low microsatellite instability(MSI-L) or proficiency mismatch repair genes (pMMR) (4, 7, 8). The results of the clinical trial IMblaze370 have underlined the challenge of immunotherapy in patients with microsatellite-stable metastatic colorectal cancer (9).

The significant effect of anti-PD-1 antibody treatment in patients with MSI-H colon cancer can be explained by its high tumor mutation burden (4–6). However, the tumor mutation burden alone cannot fully explain the lack of response of MSS to PD-1 antibodies (10, 11). Compared with other types of cancers, including cervical cancer, hepatocellular carcinoma, breast cancer and so on, that have similar mutation burden observed in MSS colon cancer patients, the anti-PD-1 antibody response rate is between 5.7%and 61.7% (10), indicating that there may be other factors that influence the immunity response. Our previous studies showed that bone marrow–derived myofibroblasts (BMFs)-rich tumors had relatively poor response to PD-L1 blockade immunotherapy and provided a novel insight into the mechanism tumor resistance to immunotherapy (12). Angiogenesis is critical in the process of colon carcinogenesis. One of the most important factors for angiogenesis is the VEGF/VEGFR axis.VEGF secreted by cancer cells stimulates the proliferation and survival of endothelial cells leading to change of vascular permeability and neo-angiogenesis (13).VEGF can have a direct effect on multiple immune cells, including T cells, regulatory T cells, dendritic cells, and myeloid derived suppressor cells (14). There are two major approaches for targeting VEGF/VEGFR axis as anti-cancer therapies: the neutralization antibody of VEGF (monoclonal antibodies) and the small molecule inhibitors that block the activity of VEGFR kinases. Bevacizumab is the most successful antibody for neutralizing VEGF.

The combination of immunotherapy with anti-angiogenesis agents is one of the many strategies currently under investigation to improve tumor response to immunotherapy (15). Fruquintinib (HMPL-013) is a small molecule inhibitor with strong potency and high selectivity against VEGFR1, 2, and 3 and was approved for the third line therapy in metastatic colon cancer in China. Fruquintinib is currently being registered in clinical study for the third line therapy in metastatic colon cancer outside China (16, 17). It has good safety and effectiveness in clinical patients. However, it is not known whether combining fruquintinib and anti-PD-1 therapy could enhance its effectiveness in CRC. The effect of the combination of fruquintinib and anti-PD-1 in MSS colon cancer has not been examined and the detailed mechanism of action has not been elucidated. The REGONIVO study found that nivolumab plus regorafenib had an ORR of 36% and a median PFS of 7.9 months in 25 patients with metastatic colorectal cancer who progressed after standard therapies (18). Our previous results have indicated that the MDSCs are important in the early stages of gastric carcinogenesis because of its pro-inflammatory role (19).

Here, we hypothesized that the combination of anti-PD-1 treatment with fruquintinib could enhance the antitumor immune response in MSS CRC. We explored whether fruquintinib could synergize with anti-PD-1 in MSS CRC by reducing angiogenesis and improving the tumor immune environments.

## Materials and Methods

### Cells and Culture Conditions

The MC38 and CT26 murine colorectal cancer cells and HUVECs were from the American Type Culture Collection (ATCC). These cell lines were cultured in complete RPMI-1640 medium, supplemented with 10% fetal bovine serum, 2mM L-glutamine, 1% (v/v) penicillin/streptomycin and maintained at 37°C in 5% CO_2_.

### Animals

The 6-week-old female C57BL/6J and BALB/c mice were purchased from Shanghai Laboratory Animal Center (Shanghai, China). Animal experiments were approved in accordance with the guidelines issued by the Animal Ethics Committee of Ruijin Hospital, Shanghai Jiaotong University School of Medicine (Shanghai China).

### Tumor Models

For the subcutaneous tumor mouse models, 1 x 10^6^ MC38 or CT26 colorectal cancer cells were resuspended in 200ul of serum-free RPMI-1640 medium and injected subcutaneously into the right flank of C57BL/6J and BALB/c mice, respectively. Six or eight days after MC38 and CT26 cell inoculation, the tumor-bearing mice were randomly assigned to four or six groups (n = 5-6 per group) according to mean tumor volume. Tumor volumes were monitored every three days with a Venire caliper, and the body weight of mice was monitored every three days. Tumor volumes were calculated using the formula (length x width x width)/2. All data were represented as means± SD.

### Drugs Information and Treatments

Fruquintinib (HMPL-013) was a gift from Hutchison MediPharma Limited (Shanghai, China). The anti-PD-1antibody- Sintilimab (cat: 11430) was a gift from Innovent Biologics Suzhou, China. CMC-Na (carboxymethyl cellulose–Na solution) was purchased from Sangon Biotech (Shanghai, China) and 0.5%CMC-Na (w/v) dissolved in sterilized water was used for oral gavage (po. 200ul every day, total 21 days) as control treatment. Fruquintinib was completely dissolved in 0.5%CMC-Na and was gavaged as 2.5mg/kg,5mg/kg or 10mg/kg (200ul every day, total 21 days); Sintilimab, an anti-PD-1 antibody, provided by Innovent Biologics, was diluted to 2.5mg/kg (200ul,twice a week, total two weeks).The treatment was began at day 8.or day 6 after tumor cell inoculation.

### Single Cell Suspensions

Tumor tissues were washed in phosphate buffered saline (PBS), minced into small fragments and then incubated in collagenase solution (1 mg/mL collagenase IV obtained from Sigma in PBS) and DNase (500ug/ml) at 37°C for 30 min. The spleen and peripheral blood were homogenized and passed through a 70 µm cell strainer to achieve single cell suspensions. Red blood cells were lysed.

### Flow Cytometry Analysis

Single-cell suspensions were incubated with anti-mouse CD16/32 antibody (clone 93; Biolegend, San Diego, CA) for 5 minutes prior to staining for immune cell markers for 15 minutes at room temperature. The following monoclonal antibodies were used: APC-CY7 conjugated anti-mouse CD45 (BD Biosciences Cat:557659 Clone:30-F11), FITC conjugated anti-mouse CD11b (Biolegend Cat:101205 Clone:M1/70), APC conjugated anti-mouse Gr1(Biolegend Cat:108412,Clone: RB6-8C5), FITC conjugated anti-mouse CD8a(Biolegend Cat:100706 Clone:53-6.7), PE conjugated anti-mouseCD4(BD Biosciences Cat:553048 Clone: RM4-5), APC conjugated anti-mouse PD-L1(Biolegend Cat:124311 Clone: 10F.9G2), and IFN-γ (XMG1.2).The flow cytometry analyses were performed using a BD Fortessa Flow Cytometer (BD Fortessa). BD FACS Diva software V.5.0.1 (BD) or Flow Jo (Tree Star) was used for data processed. For cytokine staining, harvested cells were incubated in RPMI-1640 medium with cell activation cocktail with brefeldin A (Bio legend) for 6 hours at 37°C, and stimulated cells were stained as described above.

### Immunohistochemistry and Immunofluorescence

Resected tumor tissues were fixed in 4% paraformaldehyde or embedded either in paraffin or in optimal cutting temperature compound and frozen. Vascular endothelial staining was performed using anti-CD31 antibody (clone DIA-310; for immunofluorescence [IF]), pericytes were identified with antibody against α-smooth muscle actin (SMA) (Sigma-Aldrich), and anti-rabbit secondary antibodies (Jackson ImmunoResearch) as appropriate. Cell nuclei were identified with 4′,6-diamidino-2-phenylindole (DAPI; Thermo Fisher Scientific). The total number of vessels and pericyte-covered vessels were counted in five random fields using a 200x magnification lens. Organoids (tumor tissues) were fixed in 4% formaldehyde for 15 minutes. Then, organoids were washed 3 times with PBS and immunostained. Primary antibodies targeting CD8a (1:600; Cell Signaling Technology cat: 98941), were added to the sections, which were incubated at 4°C overnight. Then, HRP-conjugated secondary antibodies (Dako, K4003), were applied to the corresponding primary antibody. Afterward, we stained the samples with diaminobenzidine (DAB; CST8059) and hematoxylin for nuclei staining. IF images were analyzed using a microscope (Leica),.

### The Combination of Fruquintinib and Sintilimab for the Treatment of CRC Patients

Five patients with refractory mCRC were given fruquintinib (3mg orally, once daily for 3 weeks, followed by 1 weeks off in a 4-week cycle) and sintilimab (200mg intravenously, once every 3 weeks). Before treatment, peripheral blood samples were collected and next-generation sequencing was performed to detect the gene profile of patients. Each of the patients signed an informed consent before the therapy. All the patients treated were not part of a prospective clinical trial.

### Statistical Analysis

Data are presented as Mean ± SD. One way ANOVA with Turkey multiple comparison post-test and the Student’s t- test were used for statistical comparisons using Graph PadPrizm8.Statistical significance was determined at the < 0.05 level (*p<0.05, **p< 0.01, and ***p<0.001, or ^#^p<0.05, ^##^p< 0.01, and ^###^p<0.001). Survival was defined as the time from treatment to death of mice due to excess tumor burden.

## Results

### Fruquintinib Inhibits CRC Tumor Growth in a Dose-Dependent Manner

**Figure 1:**
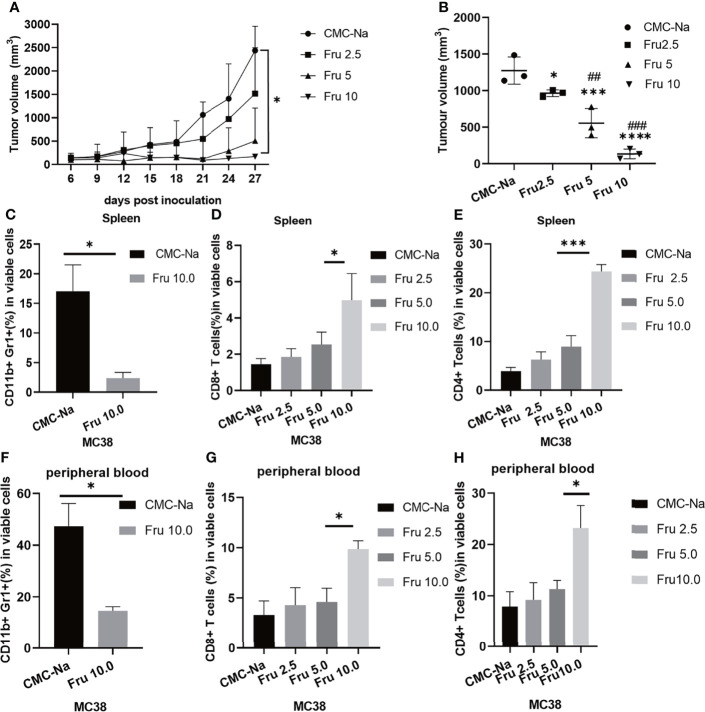
Fruquintinib inhibits tumor growth in mouse CRC allograft tumor models. Tumor-bearing mice were treated with different doses (2.5, 5, and 10mg/kg) of fruquintinib or CMC-Na control by oral gavage daily for a total of 21days. Tumor volume and immune cells were analyzed on days 14 after fruquintinib treatment (n = 3 per group). **(A)** The tumor growth curve of MC38 tumor-bearing mice (p = 0.026). **(B)** the tumor volume measured at the 28th day after treatment (p < 0.0001). **(C)** MDSCs were analyzed in spleen on day14 after fruquintinib treatment. **(D, E)** CD8^+^T cells and CD4^+^Tcells were analyzed in spleen. **(F)** MDSCs were analyzed in peripheral blood on day14 after fruquintinib treatment. **(G, H)** CD8^+^T cells and CD4^+^Tcells were analyzed in peripheral blood on day14 after fruquintinib treament. *p < 0.05, ***p < 0.001, ****p < 0.0001. ^##^p < 0.01,^###^p < 0.001. (* compared to control group, #compared to Fru2.5 group).

We first examined the effects of different doses of fruquintinib (Fru) on colon cancer syngeneic in the tumor mouse models. We treated the mice bearing MC38 tumor with three different doses of fruquintinib (2.5mg/kg, 5mg/kg, and 10mg/kg) every day for a total of three weeks. The results showed that fruquintinib could inhibit MC38 allograft tumor growth in a dose-dependent manner and the strongest inhibition was observed in the Fru 10mg/kg treatment group (**Figures 1A, B**). We examined immune cells of the spleen and peripheral blood in the mice with MC38 tumor, and found that the fractions of CD11b^+^Gr1^+^MDSCs (Myeloid-derived suppressor cells) were significantly reduced in the spleen (**Figure 1C**) and peripheral blood (**Figure 1F**) of mice in the Fru 10mg/kg treatment group. As the dose of fruquintinib increased, the proportion of CD8^+^ T cells and CD4^+^ T cells increases in the spleen (**Figures 1D–G**) and peripheral blood (**Figures 1E–H**) of mice. These results suggest that Fruquintinib inhibits CRC MC38 tumor growth and enhances antitumor immune response.

### Fruquintinib Enhances the Anti-Tumor Effect of Anti-PD-1 Antibody and Prolongs Survival of the CRC Mice

**Figure 2:**
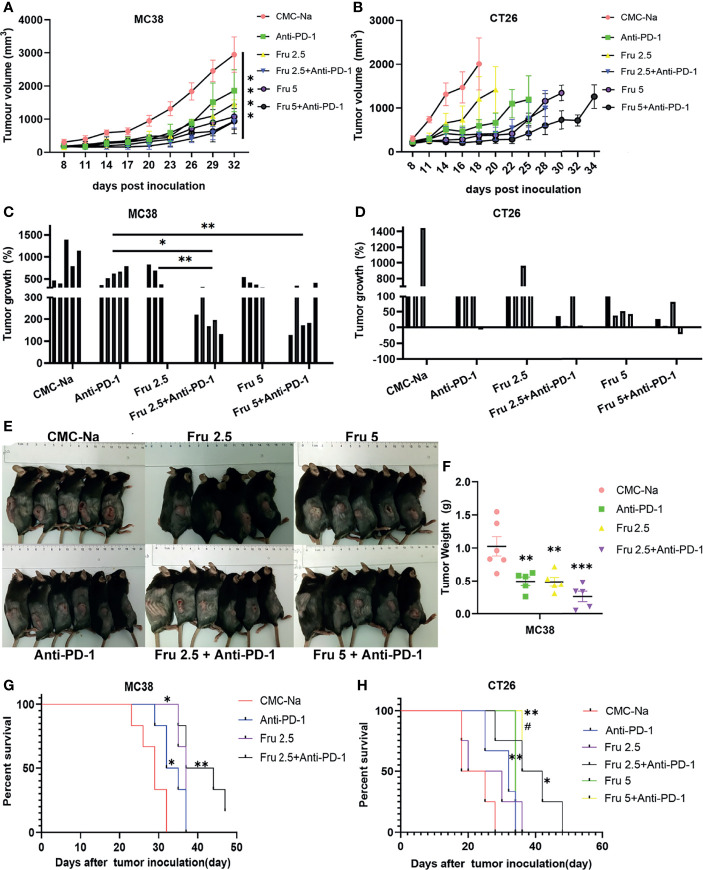
Fruquintinib enhances the anti-tumor effect of anti-PD-1 in CRC mouse models. **(A, B)** Tumor growth curve of the subcutaneously implanted tumors in MC38 and CT26 mouse models after treatment (n = 4-6). **(C, D)** Tumor volume of the subcutaneously implanted tumors in each mouse showed in 28 days after treatment **(E)** Diagram depicting the tumor volume of MC38 allograft tumor model. **(F)** Tumor weight in the MC38-bearing mice. *p < 0.05, **p < 0.01, ***p < 0.001, ****p < 0.0001 **(G, H)** Overall survival of MC38 and CT26 tumor-bearing mice. Kaplan-Meier survival curves were shown in each group of implanted tumors #p < 0.05, compared to Fru2.5 group).

Next, we determined the effects of the combination therapy on MC38 and CT26 colon cancer mouse models. Tumor-bearing mice were treated with fruquintinib at two different doses (Fru, 2.5mg/kg, and Fru, 5mg/kg) every day for a total of three weeks, sintilimab (2.5mg/kg) twice a week for a total of two weeks, or their combination. We found that single-agent treatment and combination treatment significantly inhibited tumor growth, and the combination treatment exhibited the strongest inhibition of tumor growth in the MC38 (**Figures 2A, C**) and CT26 (**Figures 2B, D**) allograft tumor models. The representative tumors from MC38 allograft tumor-bearing mice were shown in **Figure 2E**, and the tumor weight were shown in **Figure 2F**. Moreover, fruquintinib and sintilimab alone significantly increased the median survival time (MST) in MC38 (**Figure 2G**) and CT26 (**Figure 2H**) tumor-bearing mice, and the combination therapy achieved the longest MST in both models (**Figures 2E, F**).

### Combination Treatment of Fruquintinib and Anti-PD-1 Reprograms the Immune Microenvironment and Enhances Antitumor Immunity *In Vivo*

**Figure 3:**
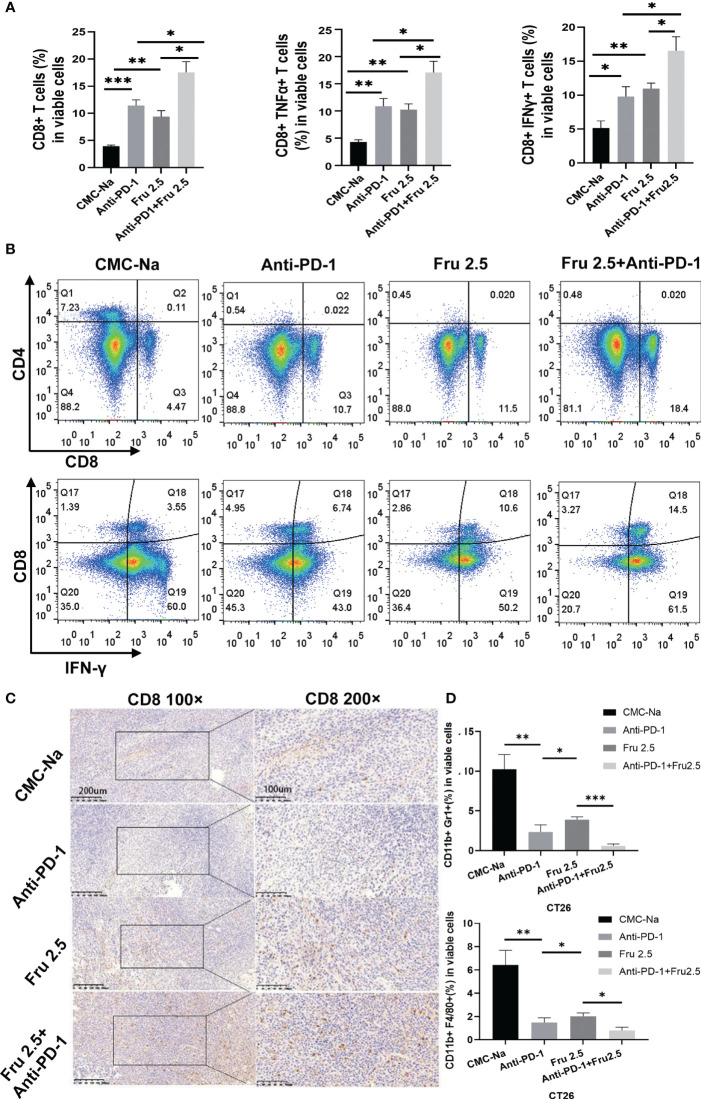
Fruquintinib enhances the effect of sintilimab to reprogram the immune microenvironment and enhance antitumor immunity in CRC. **(A)** Percentages of CD8^+^T cells, CD8^+^TNFα^+^T cells,CD8^+^IFNγ^+^ T cells in tumor tissues determined by FACS in CT26 tumor-bearing mice treated with indicated drugs (shown as fractions of CD45^+^ cells) in tumors treated with control CMC-Na, anti-PD-1, Fru2.5, arnti-PD-1 and Fru 2.5, measured by flow cytometry at day 14. **(B)** Representative images of the FACS plots of CD8^+^ and CD8^+^IFNγ^+^ CTLs. **(C)** IHC images for CD8 staining of tumor sections on day14. Scale bar, 200um. **(D)** Percentage of MDSCs (CD11b^+^Gr1^+^) and TAM (CD11b^+^F4/80^+^) in each groups on day 14. *p < 0.05, **p < 0.01, ***p < 0.001.

Next, we investigated the mechanisms by which fruquintinib enhances the effects of anti-PD-1 antibody in CRC mouse models. We first evaluated whether combination therapy increases lymphocyte infiltration. We found that the number and proportion of CD8+ T lymphocytes, CD8^+^TNFα^+^T lymphocytes and CD8^+^INFγ^+^ lymphocytes were significantly increased in tumor-bearing mice treated with fruquintinib or sintilimab alone or combination therapy (**Figure 3A**). Moreover, the combination treatment showed the strongest effect on increasing the infiltration of CD8+ T, CD8^+^TNFα^+^ T and CD8^+^INFγ^+^ lymphocytes (**Figures 3A, B**). Immunohistochemistry staining further confirmed a markedly increase of CD8^+^T lymphocytes in tumor tissues in combined treatment group (**Figure 3C**). Furthermore, treatment with the combination of fruquintinib and sintilimab significantly reduced the fraction of CD11b^+^Gr1^+^MDSCs and CD11b^+^F4/80^+^tumor-associated macrophages in CT26 allograft tumor compared to fruquintinib or sintilimab alone (**Figure 3D**). These results suggest that fruquintinib enhance the effect of anti-PD-1 to reprogram the tumor immune microenvironment and enhances antitumor immunity in CRC.

### Fruquintinib Reduces Angiogenesis in CRC Both *In Vitro* and *In Vivo*

**Figure 4:**
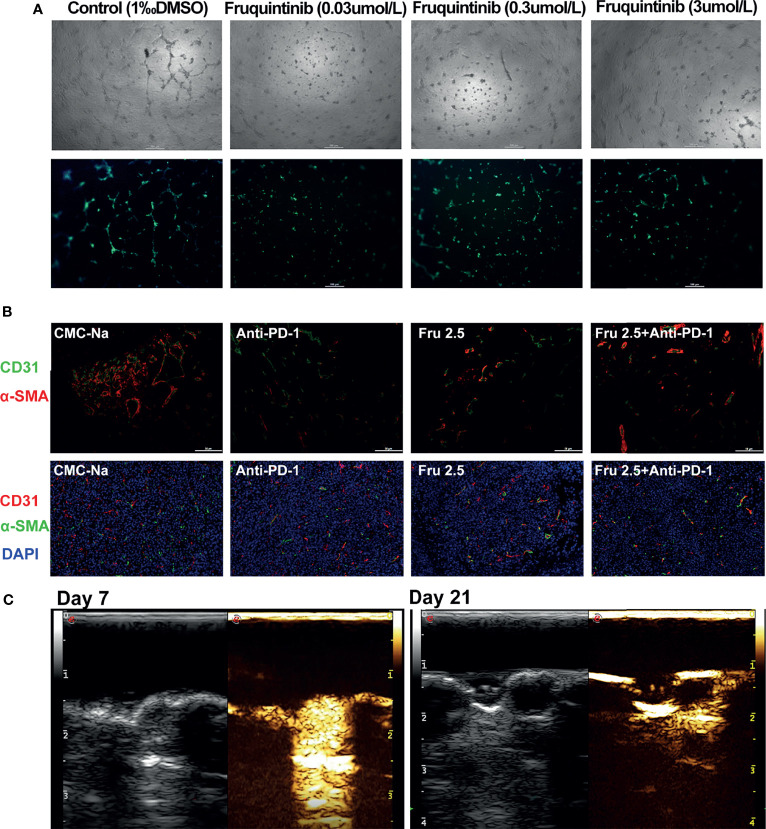
Fruquintinib reduces angiogenesis in CRC both *in vitro* and *in vivo*. **(A)** The effect of fruquintinib on tubular formation in HUVEC *in vitro* with different doses (0.03,0.3, and 3umol/l) of fruquintinib or CMC-Na control. **(B)** Representative immunofluorescent staining of sections from different treatment groups on day14. Red, CD31; α-SMA staining; green; blue, DAPI staining. **(C)** Fruquintinib reduces tumor vascular formation in CT26 colon cancer-bearing mice by angiography using ultrasound machine on day 7 and day 21 after treatment.

Previous studies suggested that fruquintinib could inhibit tubular sprouting and prevent angiogenesis *in vitro* (16). We investigated the effect of fruquintinib on tubular formation in HUVEC *in vitro*. Fruquintinib suppressed the tube branching in a dose-dependent manner in HUVEC (**Figure 4A**). The tubule length of HUVECs significantly decreased in the fruquintinib treatment group compared to the control group. In addition, we investigated the changes of intratumoral vessels in CT26 mouse model. We treated the tumor-bearing mice with low-dose of fruquintinib (2.5mg/kg, thereafter referred to as “Fru 2.5”) or 0.5% CMC-Na as a control *via* oral gavage 14 days after tumor cell inoculation, and then evaluated the tumor vasculature. The dual staining of alpha-smooth muscle actin (a-SMA) and CD31 showed that tumor vessels were significantly less in the Fru 2.5 group compared with the control group in the CT26 allograft tumor model (**Figure 4B**). Furthermore, we assessed the tumor vessels by angiography in ultrasound machine on the 14th day after treatment. SonoVue was injected into the tail vein of mice (n=3) to display the mouse tumor vascular perfusion. We found that fruquintinib reduced tumor vascular formation in CT26 colon cancer-bearing mice (**Figure 4C**).

### Safety of the Combination of Fruquintinib and Anti-PD-1 *In Vivo*

**Figure 5:**
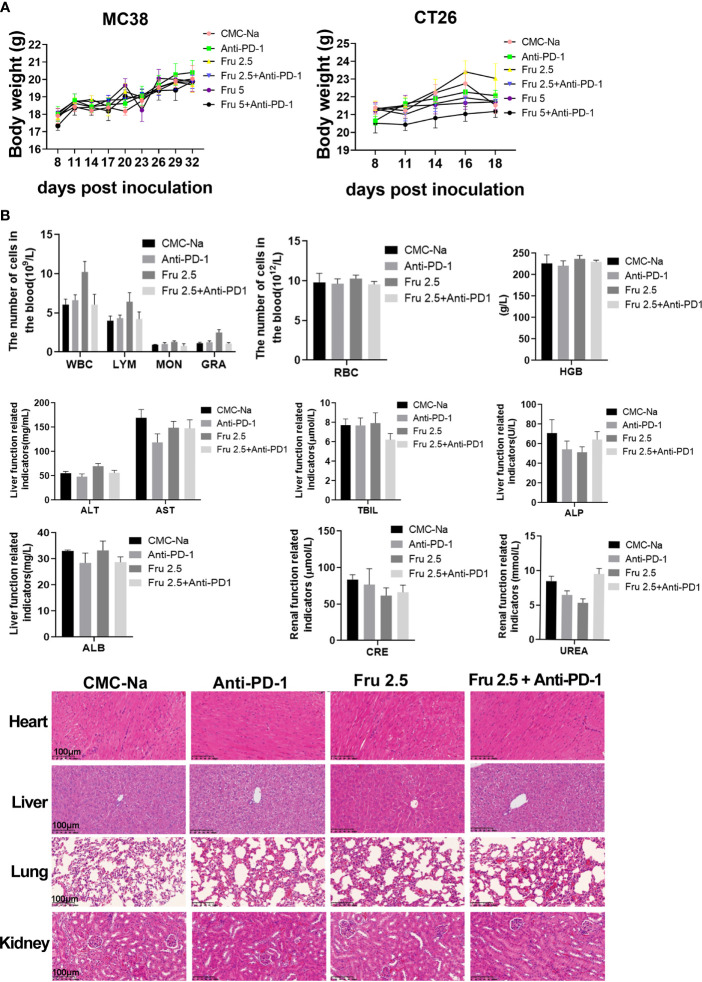
A safety of the combination of fruquintinib and anti-PD-1 *in vivo*. **(A)** Body weight of tumor-bearing mice in each group. The weight was measured every three days. **(B)** Blood cell analysis,liver and kidney function and histopathology morphology of the murine major organs (heart, liver, lung, kidney) in the mouse models.

We further determined the safety of the combination of fruquintinib and sintilimab *in vivo*. No toxicity, allergy or cachexia was observed in the tumor-bearing mice treated with the combination of fruquintinib and sintilimab. There was no significantly decreased in the body weight (**Figure 5A**). Blood cell analysis,liver and kidney function and histopathological analysis showed that no obvious damage in the murine major organs, such as the heart, liver, lung, and kidney (**Figure 5B**). These results suggest that fruquintinib in combination with sintilimab is well tolerated.

### The Combination of Fruquintinib and Anti-PD-1 Shows Antitumor Activity in Refractory Advanced CRC Patients

**Figure 6:**
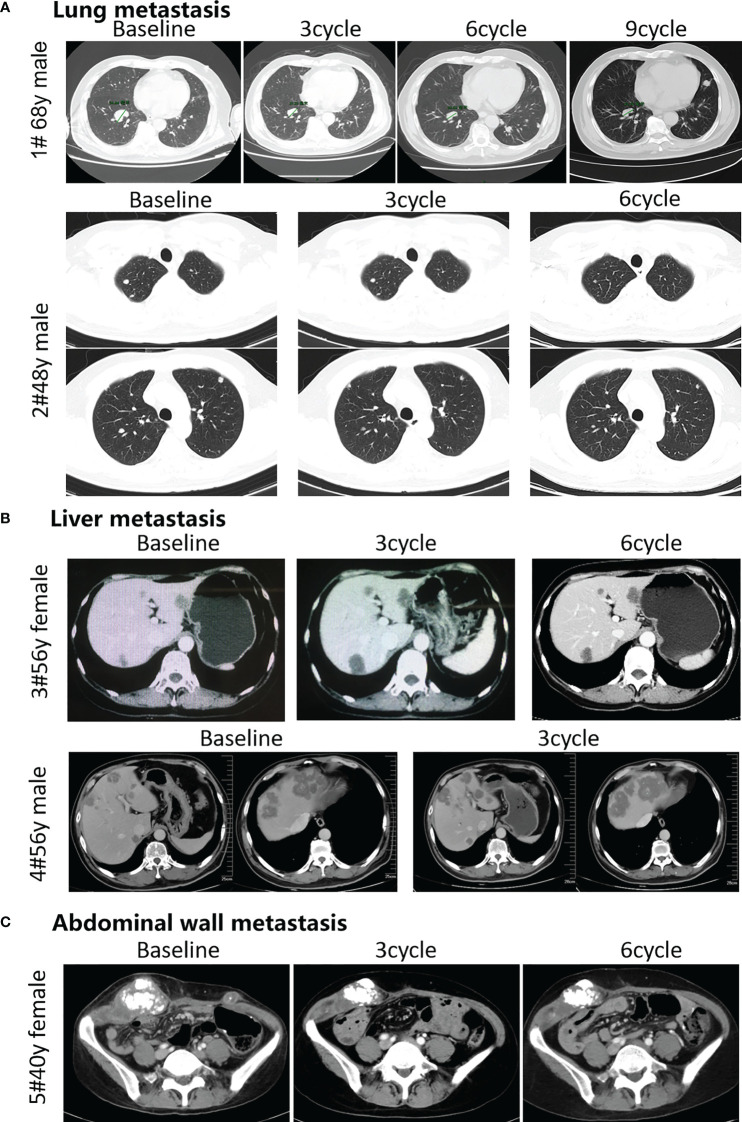
Efficacy of the combination therapy of fruquintinib and anti-PD-1 in patients in MSS mCRC. Radiologic evidence of the 5 patients who achieved PR or SD after the combination treatment. CT scans were performed at the baseline and subsequent treatment cycles. **(A)** Two CRC patients with lung metastasis: (case 1 # and case 2) were treated with anti-PD-1 and Fruquintinib, and the lesions were significantly reduced (PR). **(B)** Two CRC patients with liver metastasis: (case 3and case 4) were treated with anti-PD-1 and fruquintinib, the liver metastases showed stable morphological changes, the density became uniform and reduced, the edge became clear, and the peripheral edge enhancement disappeared. **(C)** One CRC patient with abdominal wall metastasis (case 5) were treated with anti-PD-1 and fruquintinib, and the lesions were significantly reduced (PR). Individual clinical outcomes and the 5 patients enrolled by therapeutic responses (assessed via RECIST v1.1) in **Table 1**. No, patient number; y, years old; PR, partial response; SD, stable disease; PD, progressive disease.

Based on our preclinical data from the animal experiments, we observed the effect of the combination of fruquintinib and anti-PD-1 on 5 patients with metastatic colorectal cancer who failed in more than two lines of standard treatment. Among the 5 patients, all are MSS mCRC, 2 cases CRC patients with lung metastasis, 2 cases with liver metastasis, and 1 case with abdominal wall metastasis (see **Table 1** in details). We assessed the efficacy of treatment every 3 cycles. The longest follow-up time for the patients was 18 weeks. Two CRC patients with lung metastatic lesions and one CRC with abdominal wall metastasis achieved partial response (PR). Two CRC patients with liver metastatic lesions showed stable disease (SD). The total disease control rate (DCR) was 100% (**Figure 6A**). All the included patients experienced at least one treatment-related adverse event (AE). The majority of these AEs were mild and tolerable. All patients were still on treatment and alive at the time we prepare this manuscript. More CRC patients are being recruited in the clinical study.

**Table 1:** The summary of outcomes for all treated patients.

NO.	Age	Metastasis size	Efficacy
Case1	**68y**	lung metastasis	**PR**
Case2	**48y**	lung metastasis	**PR**
Case3	**56y**	liver metastasis	**SD**
Case4	**56y**	liver metastasis	**SD**
Case5	**40y**	abdominal wall metastasis	**PR**

Individual clinical outcomes and the 5 patients enrolled by therapeutic responses (assessed via RECIST v1.1). No, patient number; y, years old; PR, partial response; SD, stable disease; PD, progressive disease.

## Discussion

Immune checkpoint inhibitors have shown good anti-tumor effects since they were applied to clinical use, especially in solid tumors such as melanoma, renal clear cell carcinoma and liver cancer (20–22). Anti-PD-1 antibodies have achieved significant effects on advanced colon cancer with high microsatellite instability (MSI-H) or missing mismatch repair genes (dMMR) (4–6), but its response rate for 80%-90% of low microsatellites (MSI-L) or mismatch repair gene wild (pMMR) colon cancer is limited (4).The clinical trial IMblaze370 showed no improved overall survival with atezolizumab plus cobimetinib or atezolizumab versus regorafenib. The results of IMblaze370 have underlined the challenge of extending the benefit of immunotherapy to CRC patients whose tumors have low baseline levels of immune inflammation, such as those with microsatellite-stable metastatic colorectal cancer (9).Optimized combinatorial strategies could expand the use of anti-PD-1 based immunotherapy in colon cancer (23–25). One of the combinatorial strategies is anti-angiogenesis drug, with the basic theory of blood vessel normalization can optimize the tumor immune microenvironment and may synergize with immunotherapy to enhance anti-tumor immunity (26).These effects remain unclear in MSS mCRC, a disease where several anti-angiogenesis drugs (fruquintinib and regorafenib) are standard treatment (17, 27). The potential of anti-angiogenesis drugs to enhance antitumor immunity in combination with immunotherapy in patients has been postulated by our preclinical data and clinical trials of others (18).

In this study, we explored the effect of the anti-angiogenesis drug fruquintinib in combination with anti-PD-1 antibody for the treatment of colon cancers, particularly in MSS CRC. Based on the past research results, we have chosen clinically relevant mouse models using the CT26 and MC38 mouse colon cancer cells, that represent for MSS CRC and MSI CRC, respectively (28, 29). We addressed one critical issue that fruquintinib can be successfully combined with anti-PD-1 to increase its efficacy in both MSI and MSS CRC. For MSS CRC, this question is in time and is in line with promising phase 1b clinical trial data in CRC patients, which showed promising high response rates in a phase1 trial (18). Based on the research that antiangiogenic agents demonstrate dose- and time-dependent influence on tumor vasculature (30, 31), we investigated the effects of distinct doses of fruquintinib on intratumoral vessels and immune components ahead of the experiments for combination treatment. We observed a favorable proinflammatory microenvironment for immunotherapy 14 days after low-dose (2.5mg/kg) fruquintinib immunotherapy treatment. Similarly, earlier studies stated that anti-angiogenesis had immune modulatory effects and combinational strategy might help to overcome resistance to anti–PD-1/PD-L1 (32, 33). We thus hypothesized that a low dose of fruquintinib might be more effective when combined with anti-PD-1immunotherapy. The work presented here is a proof that under optimal conditions, PD-1 blockade combined with antiangiogenic agents, such as fruquintinib can induce enhanced therapeutic effect. Our results from *in vivo* combination treatment experiments confirmed this hypothesis.

We showed that fruquintinib therapy not only reduced angiogenesis but also increased the proportion of T lymphocytes in our size-matched mouse tumor samples. These combination treatment interactions led to reprogramming of the immune microenvironment of CRC. Furthermore, we showed that fruquintinib not only reduced angiogenesis, but also mediated antitumor immunity by influencing lymphocyte ratio and eliminate immunosuppressive cells, such as MDSCs and TAMs. Our findings were in consistent with several previous reports in various tumor models (31, 34, 35).

In addition, we evaluated the toxicity and safety of fruquintinib, no major toxicity was found in the mouse models. Moreover, we retrospectively analyzed 5 patients with MSS mCRC, the toxicity of fruquintinib was tolerable. One of the patients’ PFS was 18 months when the data were collected. Our findings demonstratted that low-dose fruquintinib reduced immunosuppressive components and enhanced tumor immunotherapy. The preliminary clinical results further support our hypothesis. Our study is limited by using the mouse models to mimic the human TME and the study of the patient’s therapy is not a prospective clinical trial. More animals experimental systems and prospective clinical trials for further evalution are warranted.

In summary, our results suggested that fruquintinib, a VEGFR1, 2, and 3 inhibitor, when administrated at a lower dose, could optimize the immunosuppressive TME and increase the therapeutic response to immunotherapy both in mice colon cancer models and in clinical colon cancer patients. Further evaluation in randomized clinical trials are warranted.

## Data Availability Statement

The original contributions presented in the study are included in the article/**Supplementary Material**. Further inquiries can be directed to the corresponding authors.

## Ethics Statement

The patients/participants provided their written informed consent to participate in this study. The animal study was reviewed and approved by The Animal Ethics Committee of Ruijin Hospital, Shanghai Jiaotong University School of Medicine (Shanghai China). Written informed consent was obtained from the individual(s) for the publication of any potentially identifiable images or data included in this article.

## Author Contributions

ST designed the study, analysized data and revised the manuscript. QL carried out the most of experiments, analysized original data and drafted the manuscript. XC collected and analyzied the samples and the clinical data. JW collected the clinical data and revises the manuscirpt.YT, FL, BZ, and TH participated the coordination of research. CZ drafted the manuscript. All authors contributed to the article and approved the submitted version.

## Funding

The project was supported by National Science Foundation of China (NSFC 81773259,Chinese Society of Clinical Oncology Foundation (Y-Q201802-073, Y-XD202001-0318), the Science and Technique Foundation of Henan Province (No. 202102310121 for JW), Medical Science and Technology Co-construction Project of Henan Province (No. LHGJ20200167).

## Conflict of Interest

The authors declare that the research was conducted in the absence of any commercial or financial relationships that could be construed as a potential conflict of interest.

## Publisher’s Note

All claims expressed in this article are solely those of the authors and do not necessarily represent those of their affiliated organizations, or those of the publisher, the editors and the reviewers. Any product that may be evaluated in this article, or claim that may be made by its manufacturer, is not guaranteed or endorsed by the publisher.
