# Supplementary figures and images for: Fruquintinib Enhances the Antitumor Immune Responses of Anti-Programmed Death Receptor-1 in Colorectal Cancer
Source: Front Oncol. 2022 Mar 17;12:841977. doi: 10.3389/fonc.2022.841977 (PMC8968679; doi:10.3389/fonc.2022.841977)

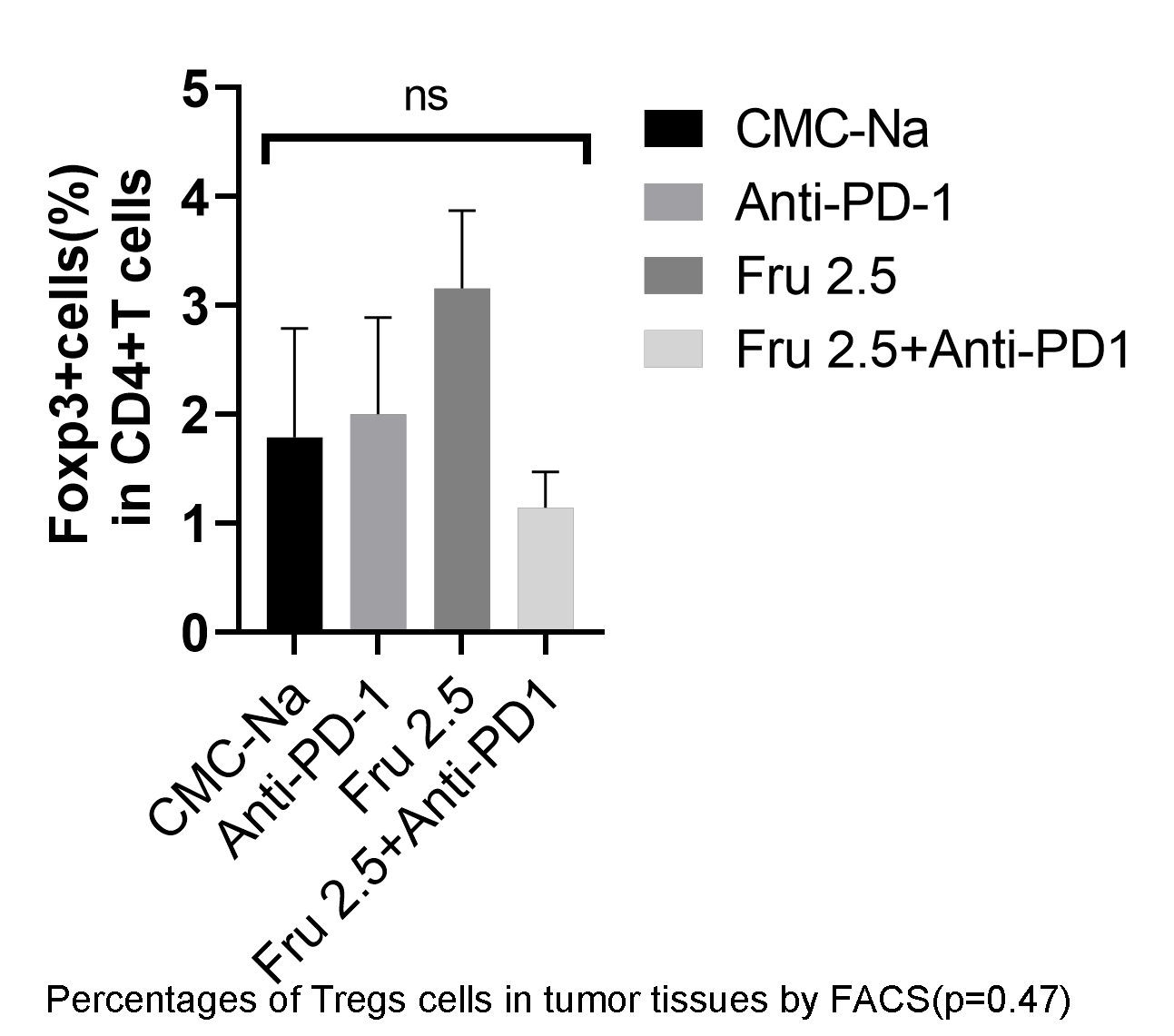

Supplement: Supplementary file 1 [file Image_1.tif]
